# Publisher Correction: The ENCODE Imputation Challenge: a critical assessment of methods for cross-cell type imputation of epigenomic profiles

**DOI:** 10.1186/s13059-025-03494-w

**Published:** 2025-02-13

**Authors:** Jacob Matthew Schreiber, Carles A. Boix, Jin wook Lee, Hongyang Li, Yuanfang Guan, Chun-Chieh Chang, Jen-Chien Chang, Alex Hawkins-Hooker, Bernhard Schölkopf, Gabriele Schweikert, Mateo Rojas Carulla, Arif Canakoglu, Francesco Guzzo, Luca Nanni, Marco Masseroli, Mark James Carman, Pietro Pinoli, Chenyang Hong, Kevin Y. Yip, Jefrey P. Spence, Sanjit Singh Batra, Yun S. Song, Shaun Mahony, Zheng Zhang, Wuwei Tan, Yang Shen, Yuanfei Sun, Minyi Shi, Jessika Adrian, Richard S. Sandstrom, Nina P. Farrell, Jessica M. Halow, Kristen Lee, Lixia Jiang, Xinqiong Yang, Charles B. Epstein, J. Seth Strattan, Bradley E. Bernstein, Michael P. Snyder, Manolis Kellis, William S. Noble, Anshul Bharat Kundaje, ENCODE Imputation Challenge Participants

**Affiliations:** 1 Department of Genetics, Stanford University, Stanford, CA USA; 2 Computer Science and Artificial Intelligence Laboratory, Massachusetts Institute of Technology, Cambridge, MA USA; 3 Department of Computational Medicine and Bioinformatics, University of Michigan, Ann Arbor, MI USA; 4 Department of Research and Development, DeepSeq.AI, San Francisco, CA USA; 5 RIKEN Center for Integrative Medical Sciences, Yokohama, Japan; 6 Department of Empirical Inference, Max Planck Institute for Intelligent Systems, Stuttgart, Germany; 7 School of Life Sciences, University of Dundee, Dundee, UK; 8 Department of Electronics, Information and Bioengineering, Politecnico Di Milano, Milan, Italy; 9 Department of Computational Biology, University of Lausanne, Lausanne, Switzerland; 10 Department of Computer Science and Engineering, The Chinese University of Hong Kong, Sha Tin, Hong Kong; 11 Sanford Burnham Prebys Medical Discovery Institute, San Diego, CA USA; 12 Department of Electrical Engineering and Computer Sciences, University of California, Berkeley, Berkeley, CA USA; 13 Department of Statistics, University of California, Berkeley, Berkeley, CA USA; 14 Department of Biochemistry & Molecular Biology, Center for Eukaryotic Gene Regulation, Pennsylvania State University, University Park, PA USA; 15 Department of Statistics, Pennsylvania State University, University Park, PA USA; 16 Department of Electrical and Computer Engineering, Texas A&M University, College Station, TX USA; 17 Altius Institute, Seattle, WA USA; 18 Epigenomics Program, The Broad Institute of MIT and Harvard, Cambridge, MA USA; 19 Department of Genome Sciences, University of Washington, Seattle, WA USA; 20 Department of Computer Science, Stanford University, Stanford, CA USA

**Correction** **:** **Genome Biol 24, 79 (2023)**

**https://doi.org/10.1186/s13059-023-02915-y**

Following publication of the original article [1], the authors identified that the affiliation 1 was incorrectly used for all authors. The correct affiliations are used in this correction article and the original article [1] has been corrected.

## References

[1] Schreiber J, Boix C, wook Lee J, et al. The ENCODE Imputation Challenge: a critical assessment of methods for cross-cell type imputation of epigenomic profiles. Genome Biol. 2023;24:79. 10.1186/s13059-023-02915-y. 37072822 10.1186/s13059-023-02915-y PMC10111747

