# Supplementary material for: The ENCODE Imputation Challenge: a critical assessment of methods for cross-cell type imputation of epigenomic profiles
Source: Genome Biol. 2023 Apr 18;24:79. doi: 10.1186/s13059-023-02915-y (PMC10111747; doi:10.1186/s13059-023-02915-y)
Supplement: Supplementary file 1 — Additional file 1. Supplementary figures and tables for follow-up and supporting analyses. [file 13059_2023_2915_MOESM1_ESM.pdf]

Additional file 1: Supplementary Tables and Figures

March 31, 2023

	team	track.rank	final.rank	teamname
1	3393580	6.568627	1	Song Lab 3
2	3388185	6.725490	2	LiPingChun
3	3393847	7.294118	3	Guacamole
4	3393817	7.784314	4	BrokenNodes_v3
5	3393574	7.843137	5	Lavawizard
6	3393860	7.882353	6	CUImpute1
7	3393861	8.803922	7	ICU
8	3393458	10.294118	8	CUWA
9	3389318	10.490196	9	Song Lab
10	3330254	10.627451	10	HLYG
11	3393417	10.921569	11	HLYG v1
12	3393457	11.294118	12	imp
13	200	11.568627	13	ChromImpute
14	3393756	11.627451	14	imp1
15	3393606	11.882353	15	BrokenNodes_v2
16	3379312	13.529412	16	CostaLab
17	3379072	13.549020	17	BrokenNodes
18	3393418	14.705882	18	HLYG v2
19	100	14.862745	19	Average
20	0	17.078431	20	Avocado
21	3393128	19.666667	21	Aug2019Imputation
22	3393851	21.156863	22	NittanyLions2
23	3344979	21.666667	23	NittanyLions
24	3393579	23.215686	24	Song Lab 2
25	3391272	24.320000	25	UIOWA Michaelson
26	3386902	25.607843	26	KKT−ENCODE−Impute

Table S1: **Overall ranking using six measures from ChromImpute.** A re-ranking of the methods used in the challenge, including ChromImpute as a baseline, using the six of the relative performance measures used to evaluate the original ChromImpute model. Ranks were computed by ranking team performance for each blind track and metric combination first, then averaging ranks and re-ranking at the track level, and finally averaging to rank overall team performance. Performance measures included (i) genome-wide correlation (GWcorr), (ii) overlap of the top 1% of imputed and observed 25 bp bins, (iii) percentage of top 1% of imputed bins in top 5% of observed bins and (iv) vice versa, and the area under the receiver operating characteristic curve (AUROC) for predicting the (v) top 1% of observed bins with imputed signal or (vi) top 1% of imputed bins using the observed signal.

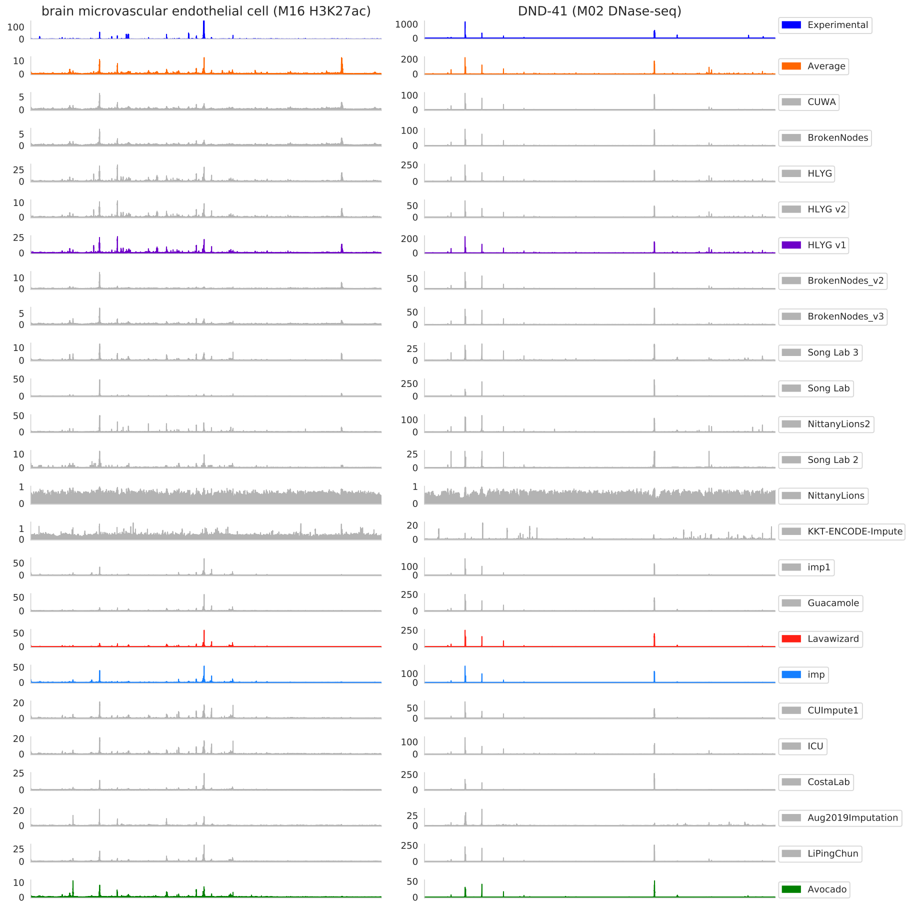

Fig. S1: **Imputations from all models at two illustrative loci.** Imputations and experimental signal for H3K27ac in brain microvascular endothelial cells and for DNase-seq in DND-41 cells. The experimental signal is proceeded by all methods, ranked by the maximum of their correlation with the average activity and with the Avocado imputations. The two baseline methods, and the winning submissions, are colored.

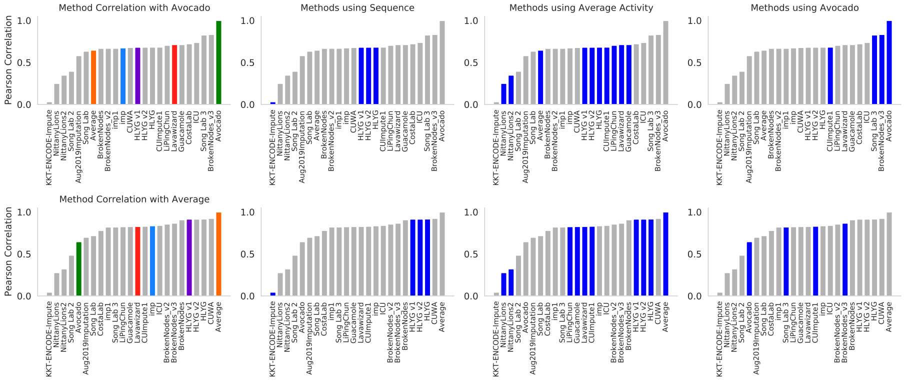

Fig. S2: **Method's correlations with the average activity and Avocado** The Pearson correlation of each method with Avocado's imputations (top row) and with the average activity (bottom row). The left column shows the baseline methods and challenge winners colored, ordered by their correlation with either Avocado or the average activity, respectively. The other panels show the same results as the left panel but color methods that use sequence features, the average activity, or Avocado's imputations, as input.

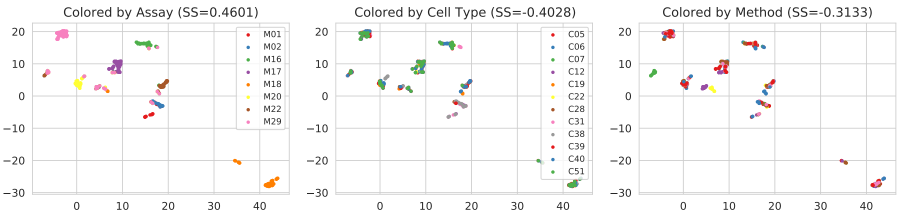

Fig. S3: **A projection of all experiments.** A UMAP projection of the experimental and imputed tracks from all teams based on Pearson correlation averaged across each chromosome. The panels are colored, and show the Silhoutte Score when clustering, by assay type, cell type, and method.

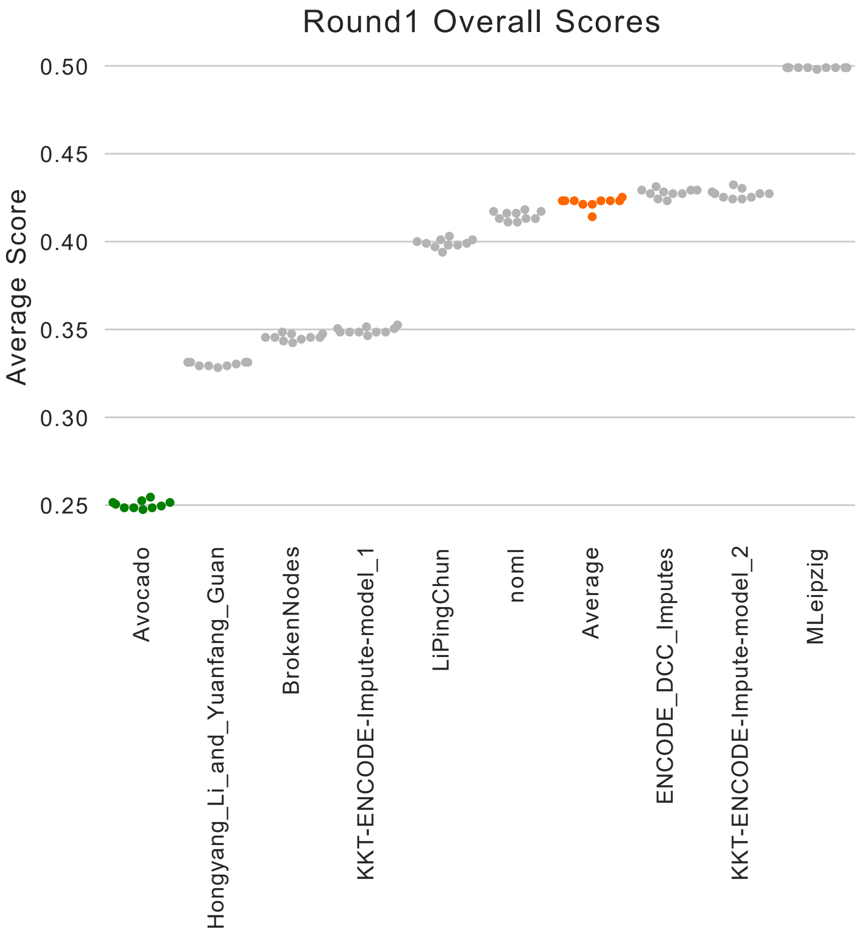

Fig. S4: **Round 1 Scores.** The final leaderboard results for each participant in round 1 of the challenge. Each dot is the score calculated on one of ten bootstraps, and the participants are ordered by overall score. Lower scores indicate better performance.

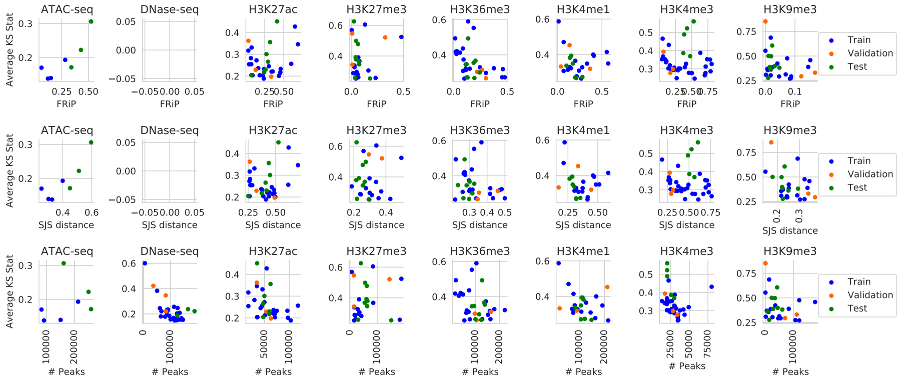

Fig. S5: **Quality control metrics for challenge experiments.** Each row displays a different quality control metric, each column displays that measure within a certain assay, and each dot in the panel corresponds to the metric score for a particular experiment. Each dot is colored based on if it is a training (blue), validation (orange), or test set (green) experiment.

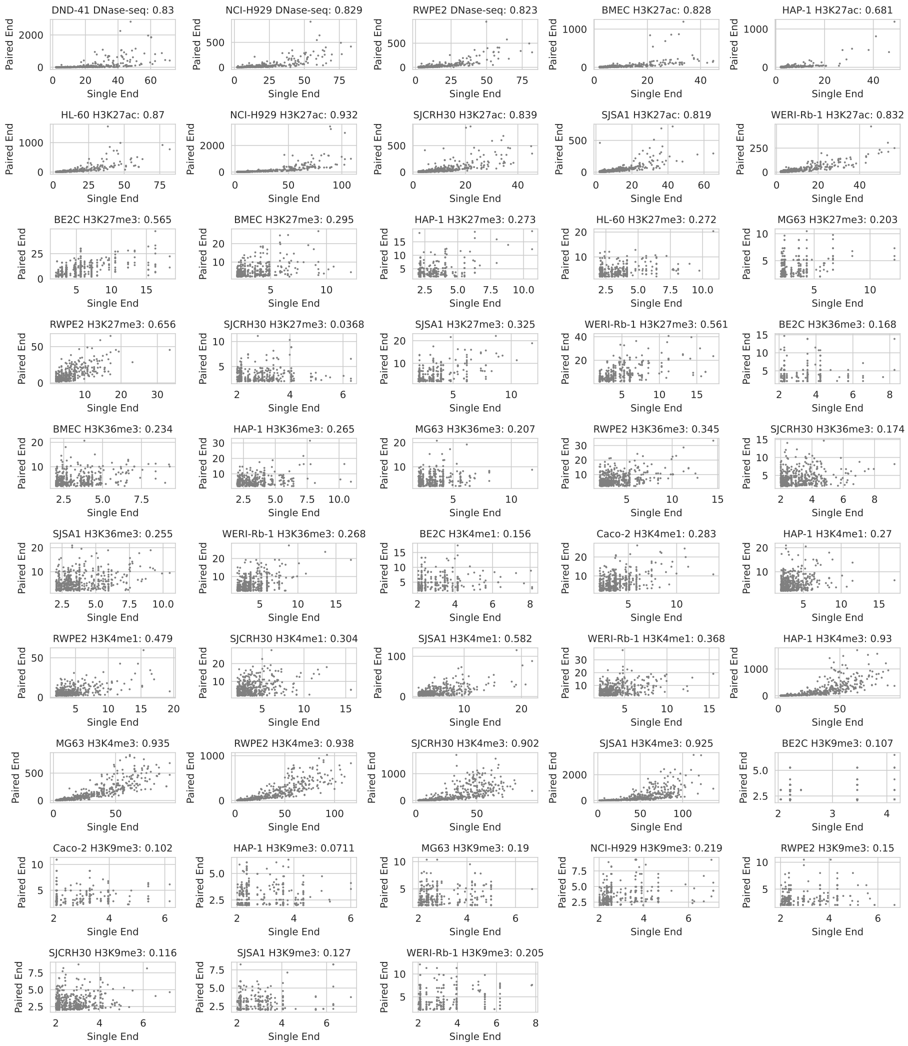

Fig. S6: **Signal values with paired-end and single-end processing** For each of the 48 reprocessed experiments, 10Mb in the center of chr1 are considered when the reads are processed using the original paired-end processing or the single-end reprocessing. Only 500 random positions within this block are visualized for each experiment, but the Spearman correlation shown for each panel is calculated across the entire 10Mb block.

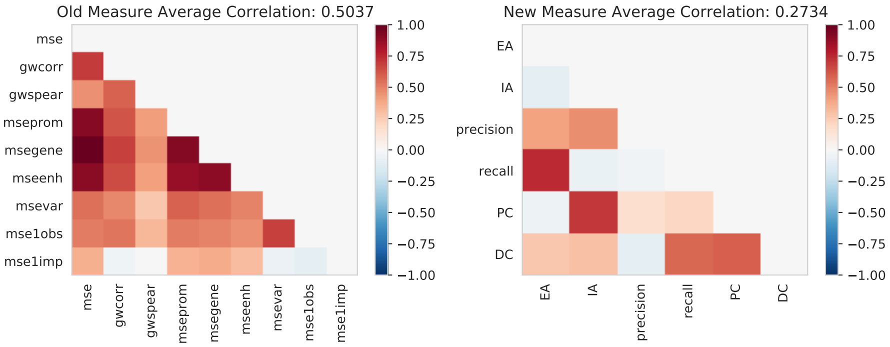

Fig. S7: **Rank correlation of performance measures** Rank correlations are calculated between each performance measure by calculating the Spearman correlation across all teams for each pair of measures. For the original nine measures (left), the rank of each team for each measure is averaged across all ten folds. For the new six measures the Spearman correlation is calculated directly on the performance values because there are no bootstraps.

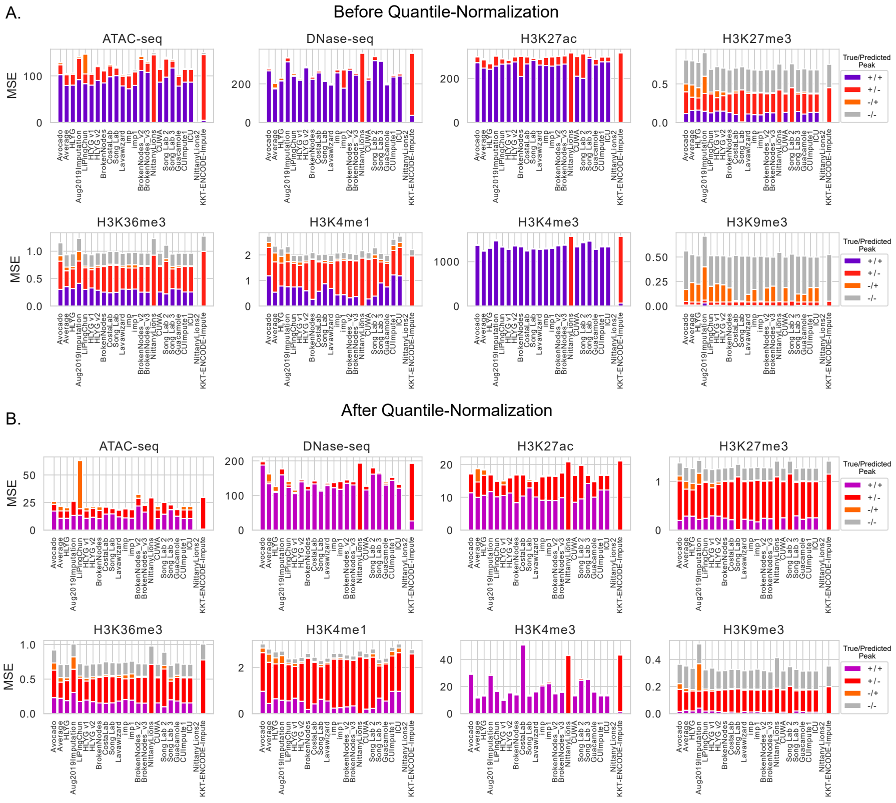

Fig. S8: **Error partitioned by peak calls** The genome-wide squared error was partitioned into four categories for each assay: within correctly predicted peaks (+/+), within true peaks incorrectly not predicted to be peaks (+/-), within background regions incorrectly predicted to be peaks (-/+), and within background regions correctly predicted to be background (-/-). True peaks were identified using the MACS2 peak caller and predicted peaks were identified as a predicted signal value ≥ 2. (A) The error for each method on the original signal. (B) The error for each method on the quantile-normalized signal.

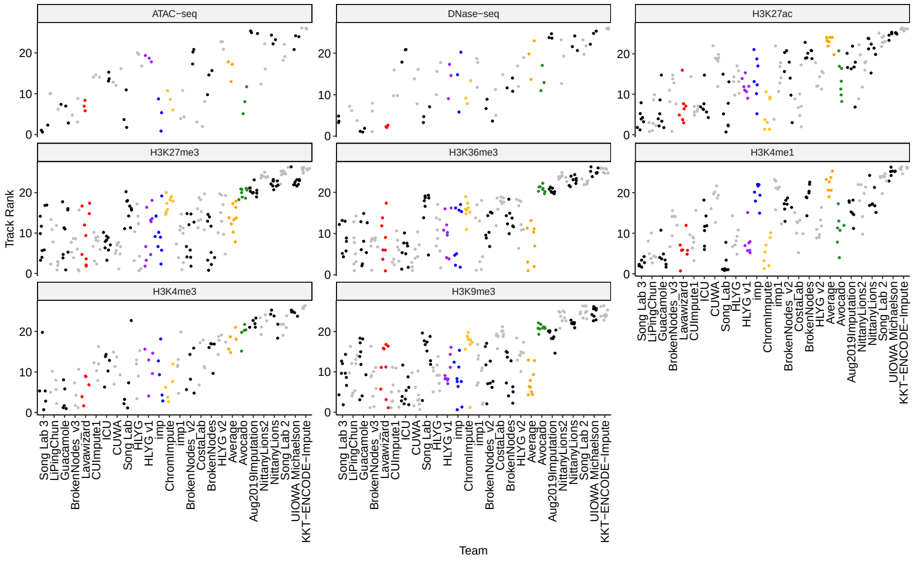

Fig. S9: **Rankings per assay using six measures from ChromImpute.** A re-ranking of the methods used in the challenge broken down by assay, with each dot representing an experiment in the blind test set. Colors alternate black and grey by team except for colors that highlight top performing teams as in the main text. ChromImpute and the average predictor are colored in yellow and Avocado in green.
